# Supplementary material for: Respiratory Syncytial Virus Matrix Protein Is Sufficient and Necessary to Remodel Host Mitochondria in Infection
Source: Cells. 2023 May 4;12(9):1311. doi: 10.3390/cells12091311 (PMC10177070; doi:10.3390/cells12091311)
Supplement: Supplementary file 1 [file cells-12-01311-s001.zip › cells-2330814-supplementary.pdf]

Supplementary Figure S1

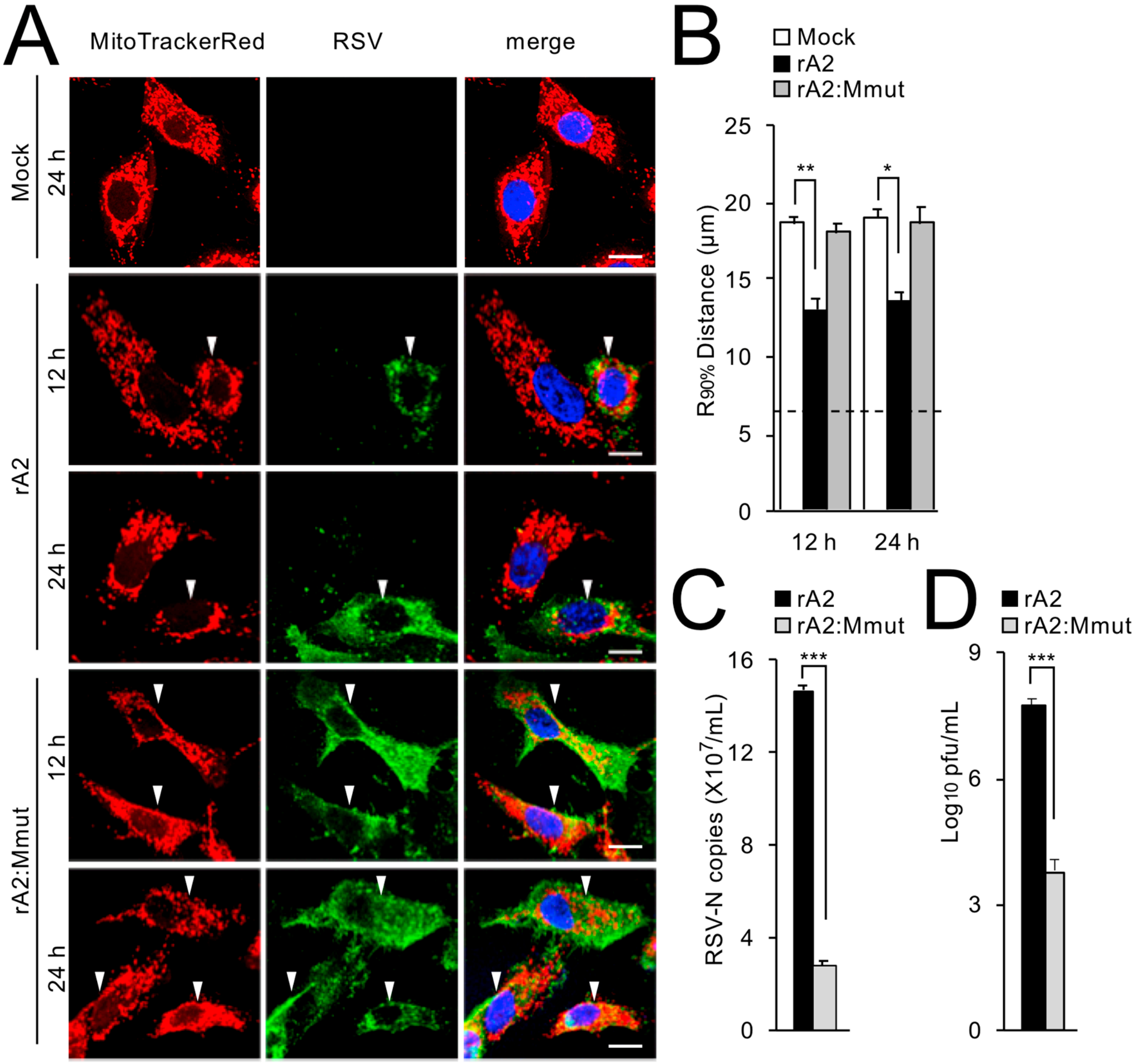

Supplementary Figure S1. Mitochondrial perinuclear clustering in infected cells is critical to RSV virus production. Vero cells were mock-, rA2- or rA2 M:R170T/K172T (rA2:Mmut)-infected (MOI 3) for the times indicated and then subjected to (**A**, **B**) fixation/immunostaining and $R_{90\%}$ analysis as per Figure 1AB, where white arrowheads denote infected cells (scale bar = 10 μm); or (**C**, **D**) quantification of cell-associated (**C**) virus genomes by qPCR, and (**D**) infectious virus by plaque assay. Results shown represent the mean ± SEM from 3 independent experiments assayed in triplicate. ***$p < 0.001$, **$p < 0.01$, *$p < 0.05$.
